# Identification of the hub genes related to adipose tissue metabolism of bovine

**DOI:** 10.3389/fvets.2022.1014286

**Published:** 2022-11-09

**Authors:** Xiaohui Wang, Jianfang Wang, Sayed Haidar Abbas Raza, Jiahan Deng, Jing Ma, Xiaopeng Qu, Shengchen Yu, Dianqi Zhang, Ahmed Mohajja Alshammari, Hailah M. Almohaimeed, Linsen Zan

**Affiliations:** ^1^College of Animal Science and Technology, Northwest A&F University, Xianyang, China; ^2^Department of Biology, College of Science, University of Hail, Ha'il, Saudi Arabia; ^3^Department of Basic Science, College of Medicine, Princess Nourah Bint Abdulrahman University, Riyadh, Saudi Arabia; ^4^National Beef Cattle Improvement Center, Northwest A&F University, Xianyang, China

**Keywords:** different diets, WGCNA, lipid metabolism, energy metabolism, hub genes

## Abstract

Due to the demand for high-quality animal protein, there has been consistent interest in how to obtain more high-quality beef. As well-known, the adipose content of beef has a close connection with the taste and quality of beef, and cattle with different energy or protein diet have corresponding effects on the lipid metabolism of beef. Thus, we performed weighted gene co-expression network analysis (WGCNA) with subcutaneous adipose genes from Norwegian red heifers fed different diets to identify hub genes regulating bovine lipid metabolism. For this purpose, the RNA sequencing data of subcutaneous adipose tissue of 12-month-old Norwegian red heifers (*n* = 48) with different energy or protein levels were selected from the GEO database, and 7,630 genes with the largest variation were selected for WGCNA analysis. Then, three modules were selected as hub genes candidate modules according to the correlation between modules and phenotypes, including pink, magenta and grey60 modules. GO and KEGG enrichment analysis showed that genes were related to metabolism, and participated in Rap, MAPK, AMPK, VEGF signaling pathways, and so forth. Combined gene interaction network analysis using Cytoscape software, eight hub genes of lipid metabolism were identified, including *TIA1, LOC516108, SNAPC4, CPSF2, ZNF574, CLASRP, MED15* and *U2AF2*. Further, the expression levels of hub genes in the cattle tissue were also measured to verify the results, and we found hub genes in higher expression in muscle and adipose tissue in adult cattle. In summary, we predicted the key genes of lipid metabolism in the subcutaneous adipose tissue that were affected by the intake of various energy diets to find the hub genes that coordinate lipid metabolism, which provide a theoretical basis for regulating beef quality.

## Introduction

Because the worldwide demand for meat products is consistently increasing (1, 2), how to produce high-quality beef has always been a topic of concern among scholars (3). RNA sequencing has widely been used in animals to mine potential regulatory molecules for many years. For instance, using this approach, researchers have identified several pathways by which KLF6 is involved in lipid metabolism (4). Studies have indicated that the variation of the energy and protein levels in feed (5), and the change in the energy and protein intake ratio (6) have a non-negligible regulatory influence on cattle growth and development, production performance, metabolic level, immune function, and reproductive capacity. Meanwhile, the content and distribution of adipose tissue which plays a role in the metabolism of meat is an important factor affecting the taste and quality of beef (7, 8). A study demonstrated that feeding a high-energy diet effectively increased fat deposition in fattening cattle (9). However, monotonous performance and phenotypic changes have prevented us from understanding the molecular mechanistic effects of different energy and protein intakes on beef-related metabolism (10). At present, the complex molecular regulatory mechanism of bovine subcutaneous adipose tissue is not clear (11). Scholars at home and abroad have predicted many key signaling pathways and regulatory genes regulating bovine lipid metabolism through molecular biology and bioinformatics analysis and other research methods (12–14).

Weighted gene co-expression network analysis (WGCNA) is currently the preferred algorithm for calculating the correlation between genes and phenotypes (15). Based on high-throughput RNA sequencing data, it relies on the R software package (16) for data analysis, constructs a cluster tree portraying different gene modules, integrates genes with the same biological function into one module systematically (17). The gene expression patterns within the module are comparable (18), when they are associated with phenotypes and participate in the same biological process (19). To sum up, it is suitable for analyzing complex regulatory mechanisms. At present, in the research of livestock and poultry, researchers mainly forecast the regulatory network of important economic traits (20), the molecular regulatory mechanism of disease occurrence (21), and the associated network between the phenotype of livestock and the internal molecular regulatory mechanism by incorporating other bioinformatics analysis tools (22, 23). Therefore, it is viable to employ WGCNA to explore hub genes and metabolic processes that alter fat deposition. At present, some results have been moderately reported in pigs (24), chickens (25), cattle (26), and other animals (27).

Here, the association analysis between subcutaneous adipose tissue genes of Norwegian red heifers fed on different energy diets was conducted to predict the hub metabolic regulatory genes of subcutaneous adipose tissue. Qinchuan beef cattle were used as the molecular research objects to verify the generality of this result, which provide a theoretical basis for regulating the metabolism of subcutaneous adipose tissue and improving beef quality.

## Materials and methods

### Sample collection and processing

Tissues from the heart, liver, spleen, lung, kidney, subcutaneous fat, and muscle from a healthy adult cattle and newborn calf were collected after slaughter, frozen immediately with liquid nitrogen, and stored at −80°C. The samples in this study were collected from healthy Qinchuan beef cattle with consistent growth and bred at the National Beef Cattle Improvement Center of Northwest Agriculture and Forestry University (Yangling, China).

### Data collection and collation

The reads count matrix of transcriptome data of each sample used in this study were obtained from GSE79347 dataset (https://www.ncbi.nlm.nih.gov/geo/query/acc.cgi?acc=GSE79347). The datasets respectively were from two types of Norwegian red heifers (high-yielding dairy group, hmy; normal milk producing group, lcm) fed with four kinds of feeds, including high energy high protein (HEHP), high energy low protein (HELP), low energy high protein (LEHP), and low energy low protein (LELP). Six biological replicates were taken from each treatment group, with a total of 48 samples.

The raw data were converted into standard fastq format through SRA tools (version 2.8.1) software, Then the quality control and preprocessing of the data were carried out using the FastQC (version 0.11.9) (https://www.bioinformatics.babraham.ac.uk/projects/fastqc/). For downstream WGCNA analysis, we first extracted the protein-coding gene-set according to gene annotation information from Ensembl database (https://asia.ensembl.org/index.html). Then, the FPKM (Fragments Per Kilobase of exon model per Million mapped fragments) value of each gene was calculated according to the reads count, which aims to normalize the gene expression.

### Weighted gene co-expression network construction

The weighted co-expression network was constructed by the WGCNA package in R Studio (28, 29). The gene expression level, first, was calculated based on the raw counts of each sample to construct a gene expression matrix of 48 samples according to FPKM (Fragments per Kilobase of transcript per million) which is a standardized measurement of transcription abundance. The top 75% (30) genes with the largest variation were selected by the gene expression level to construct a correlation matrix. Then we chose the soft threshold β that best fits the scale-free network to obtain the scale-free adjacency matrix which was computed into a Topological Overlap Matrix (TOM). We constructed a hierarchical clustering tree according to the corresponding dissimilarity (1-TOM), the minimum number threshold of genes in each module was set to 50, to identify modules by merging co-expression similarity genes. In addition, similar modules were merged based on the dissimilarity of module eigengenes with a threshold less than 0.20 (31). Finally, Pearson correlation analysis was performed between modular characteristic genes (ME) and lipid metabolism. The results of the correlation and significance levels of module eigengenes (MEs) with phenotypes were displayed by the R software package ggplot2, and the gene significance (GS) and module membership (MM) values were exported.

### Functional annotation of module genes and screening of hub genes

The Pearson correlation coefficient greater than 0.3 and *p* < 0.05 were used as thresholds to select modules for GO function annotation and Kyoto Encyclopedia of Genes and Genomes (KEGG) pathway enrichment analysis. The online tool g:Profiler (https://biit.cs.ut.ee/gprofiler/) was used for GO function annotation with default parameters (32). There were three categories of GO annotation: biological process (BP), cellular component (CC), and molecular function (MF). The results were consistent with a *p* < 0.05 arranged in ascending order of *p*-value, and the top 5 of the obtained results were displayed. The module genes of KEGG pathway enrichment analysis were implemented by KOBAS (http://kobas.cbi.pku.edu.cn/genelist/) with default parameters (33) and screening condition for significant enrichment according to *p* < 0.05.

The higher the GS value, the greater the correlation between this gene and this phenotype is; the higher the MM value is, the greater the contribution of this characteristic gene to this module; the gene with the highest GS and MM values in the module is regarded as a hub gene. Therefore, the intramodular key genes were chosen based on | GS | > 0.2, | MM | > 0.9 with a *p* < 0.05 (34). The interaction network between key genes obtained through weighted gene co-expression network analysis and its target genes were arranged in descending order of weight, and the top 200 (35) genes were selected and imported into Cytoscape_V3.8.2 software (36) to select hub genes.

### Quantitative real-time PCR analysis

After processing the beef tissues, we used RNAiso Plus Kit (Trizol, Takara, Beijing, China) to extract the total RNA from the beef heart, liver, spleen, lung, kidney, muscle, and adipose tissues. The cDNA was obtained by reverse transcription kit (PrimeScript™ RT reagent Kit with gDNA Ewraser, Takara, Beijing, China). The DNA and CDS region sequences of hub genes were downloaded from the NCBI database for primer design. Then, the designed primer sequences were uploaded to BLAST (https://blast.ncbi.nlm.nih.gov/Blast.cgi) for specificity test. Primer sequences are shown in Table 1. The relative expression levels of hub genes in adult cattle heart, liver, spleen, lung, kidney, muscle, and adipose tissue were measured, and the expression levels of hub genes in the adult cattle and the newborn calves' adipose tissue were compared. Quantitative real-time PCR were performed using the PerfectStart Green qPCR SuperMix kit (TransGen Biotech, Beijing, China), and the results were obtained. It should be noted that three biological replicates and technical replicates were performed for all experiments. SPSS 25 (37) and Graphpad Prism 9 (38) softwares were used for difference significance analysis and mapping, respectively.

**Table 1:** The hub genes' quantitative PCR primer sequences.

**Genes**	**Primer sequences** **(5** ^**′**^ **-3** ^**′**^ **)**		**Annealing temperature**
*β-actin*	F:	ATCGGCAATGAGCGGTTC	60°C
	R:	CGTGTTGGCGTAGAGGTC	60°C
*TIA1*	F:	GGATACAGCCGGAAATGATCCA	60°C
	R:	TGTGTGCTGACAACGGTACT	60°C
*LOC516108*	F:	GCTGTAGGGCGGAAGATGTG	60°C
	R:	AGCCTCCTGTCCAGAGACATA	60°C
*SNAPC4*	F:	CTTCAAGCAGTTGCCAAGTATG	60°C
	R:	CCAACGCCGTATTTTTCTATC	60°C
*CPSF2*	F:	CGCTTTGGGGCAGGACTTAT	60°C
	R:	ATAAATTCCTTCTGGGCGGGG	60°C
*CLASRP*	F:	GAAGAAGGCATCCATCGGCTACAC	60°C
	R:	GCATCCTGACGAAGTCGCCATC	60°C
*ZNF574*	F:	TACCGCAAAGCAGAAGAGG	60°C
	R:	ACCTCGGTCACCACCTCAGT	60°C
*MED15*	F:	ACGTTTCGGGGCAGGAGA	60°C
	R:	TCTTGGCCTTCAGGAACACG	60°C
*U2AF2*	F:	GTCTCGCGCAGCCTTCTTA	60°C
	R:	GAGAGGAAACGGAGAAGGGC	60°C

### Statistical analysis

The relative expression levels of different quantitative real-time PCR data were analyzed by the 2^−ΔΔCt^ method. All experiments were performed in triplicate. The results were expressed as mean ± standard error of the mean (SEM). Statistical analyzes were performed with SPSS 25 (37) and Graphpad Prism 9 (38). Differences between groups were calculated by Analysis of Variance (ANOVA) methods and significance was indicated by lowercase letters or asterisks. ^*^p < 0.05, significant; ^**^p < 0.01, moderately significant; ^***^p < 0.001, highly significant; and ^****^p < 0.0001, extremely significant.

## Results

### Construction of weighted gene co-expression network

**Figure 1:**
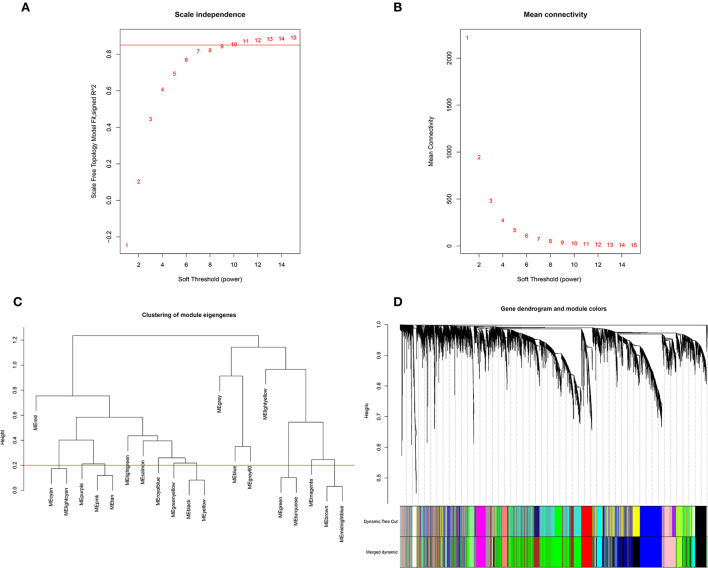
Identify soft thresholds and filter modules. **(A)** Scale-free topology fit index. **(B)** Mean connectivity of different soft-thresholding power. **(C)** The cluster dendrogram of gene modules eigengenes. **(D)** The gene clustering dendrogram.

A total of 7,630 genes, which was the largest variation, were obtained for subsequent analysis. There was no outlier in the samples through 48 samples drawn with a hierarchical clustering tree. First, the soft threshold was filtered. When the soft threshold β = 10 in this test, the scale-free network fitting index (R^2^) was greater than 0.85 (Figure 1A) and the average connectivity approached 0 (Figure 1B), which conforms to the characteristics of a scale-free network. Then, by merging similar modules with the dynamic hybrid-cutting method and setting the MEDissThres cutting line to 0.20, light cyan was merged with cyan, yellow was merged with black, turquoise was merged with green, midnight-blue was merged with brown, and tan and purple were merged with pink (Figure 1C). Finally, there were 15 modules with different colors, blue, grey60, red, cyan, pink, light-green, salmon, royal blue, black, green-yellow, light-yellow, magenta, brown, green, and grey (Figure 1D). The number of genes in the different modules had a large variation, from 85 genes in the royal-blue module to 1954 in the green module (Supplementary Table S1).

### Identification of candidate modules

**Figure 2:**
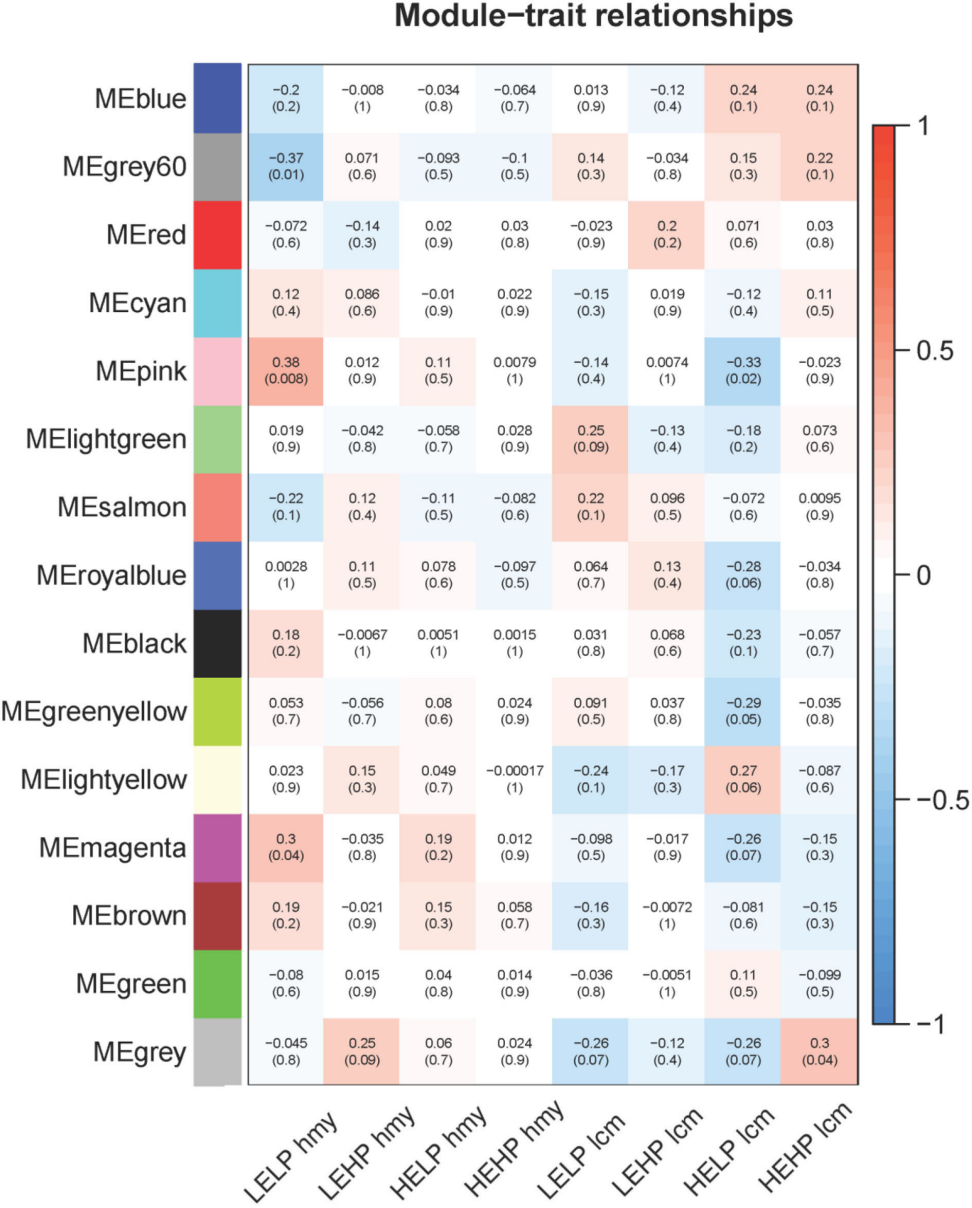
Relationship between modules and different feeding methods. The numbers on the top of the block represent the correlation and the *p*-value on the bottom. The horizontal axis represents the different feeding methods, and the vertical axis represents the eigenvector of each module. Red means positive correlation, and blue means negative correlation.

As shown in Figure 2, there were three modules among 15 modules whose filter condition | *R* | > 0.3 (*p* < 0.05) were selected as key genes candidate modules, including the grey60 module, pink module, and magenta module. The grey60 module (*R* = −0.37, *p* = 0.01) significantly negatively correlated with low energy and low protein diets. Conversely, the pink module (*R* = 0.38, *p* = 0.008) significantly correlated with low energy and low protein diets, as well as significantly negatively associated with high energy and low protein diets (*R* = −0.33, *p* = 0.02). The magenta module (*R* = 0.3, *p* = 0.04) was significantly positively related to low energy and low protein diets. According to the analysis results, the different energy intakes of Norwegian red heifers had a significant impact on their gene expression. Therefore, these three modules were screened as lipid metabolism-related modules for subsequent functional analysis and identification of hub regulatory genes.

### Functional enrichment analysis of three modules

**Figure 3:**
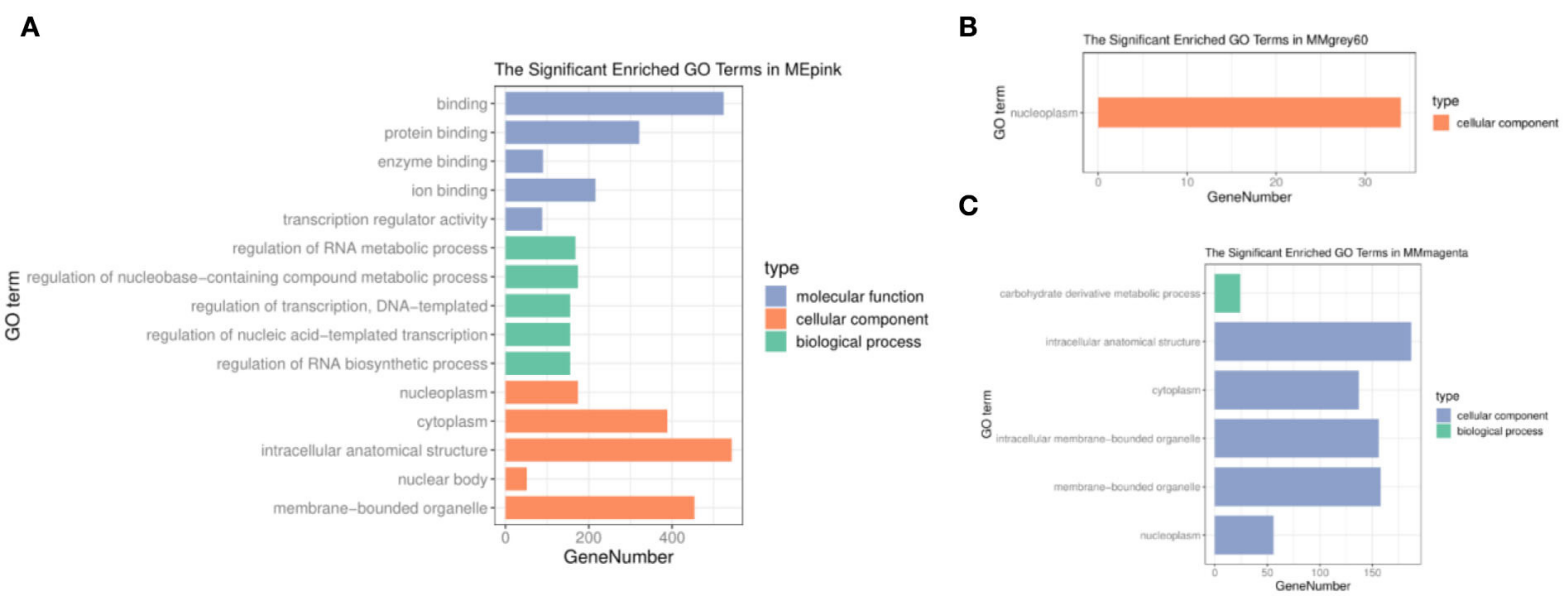
GO enrichment analysis. **(A)** Pink module. **(B)** Grey60 module. **(C)** Magenta module.

To understand the molecular functions and biological pathways of genes in co-expression modules closely correlated with different feeding methods, the genes of three modules were executed to GO and KEGG enrichment analyzes above. Among the GO terms (Supplementary Table S2), the pink module genes were mainly used as nucleoplasm, cytoplasmic, and organelle components that participated in the regulation of the RNA metabolic process, regulation of nucleobase compound metabolic process, regulation of transcription, regulation of nucleic acid–templated transcription, and regulation of RNA biosynthetic process (Figure 3A). The cellular component of the grey60 module genes was significantly enriched in the nucleoplasm (Figure 3B). Moreover, the biological processes of the magenta module were closely related to carbohydrate derivative metabolic processes (Figure 3C).

**Figure 4:**
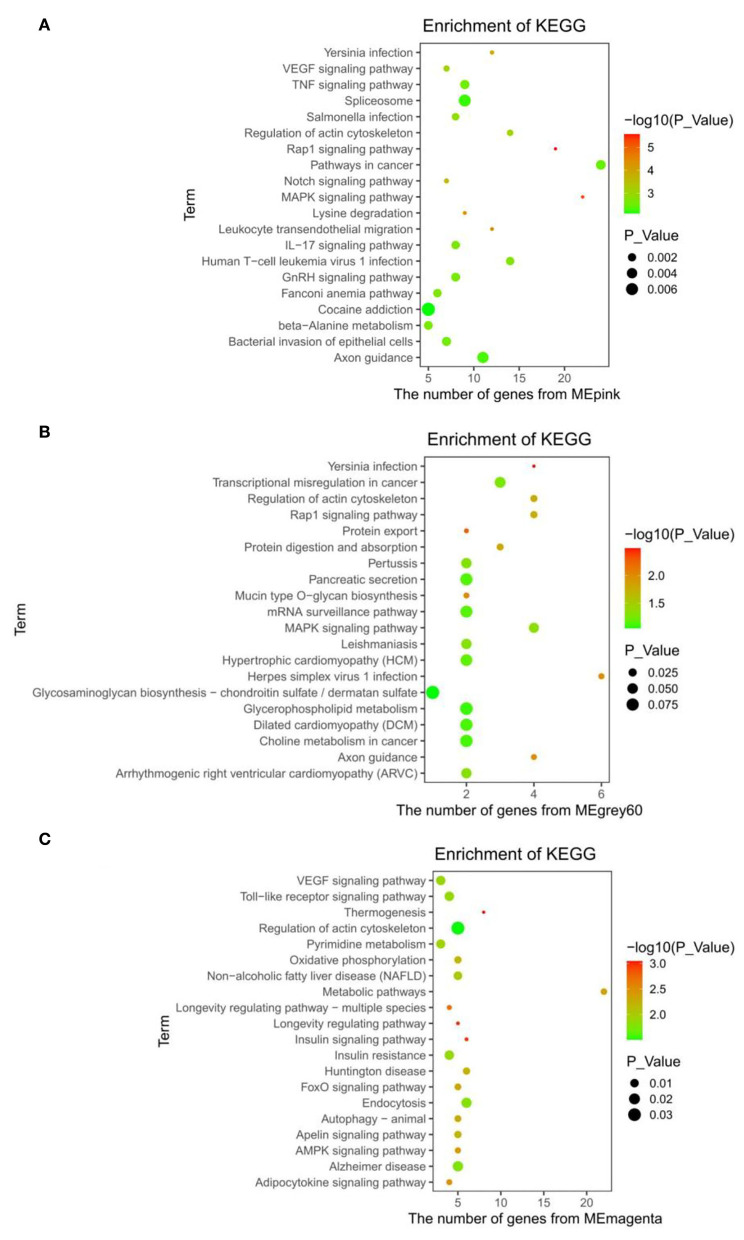
KEGG enrichment results. **(A)** Pink module. **(B)** Grey60 module. **(C)** Magenta module.

The KEGG enrichment results of the pink module showed that the pathways, such as Rap1, MAPK, Notch, VEGF, IL-17, GnRH signaling pathway, and beta-alanine metabolism were related to different energy intakes (Figure 4A). Additionally, the pancreatic secretion, glycerophospholipid metabolism, Rap1, and MAPK signaling pathway were enriched in the grey60 module (Figure 4B) and the thermogenesis process, insulin resistance process, non-alcoholic fatty liver disease (NAFLD), oxidative phosphorylation, pyrimidine metabolism, insulin signaling pathway, adipocytokine signaling pathway, AMPK signaling pathway, metabolic pathways, and VEGF signaling pathway were enriched in the magenta module and were closely associated with low energy and low protein diets (Figure 4C). The complete results are shown in Supplementary Table S3.

### Hub genes associated with lipid metabolism

**Figure 5:**
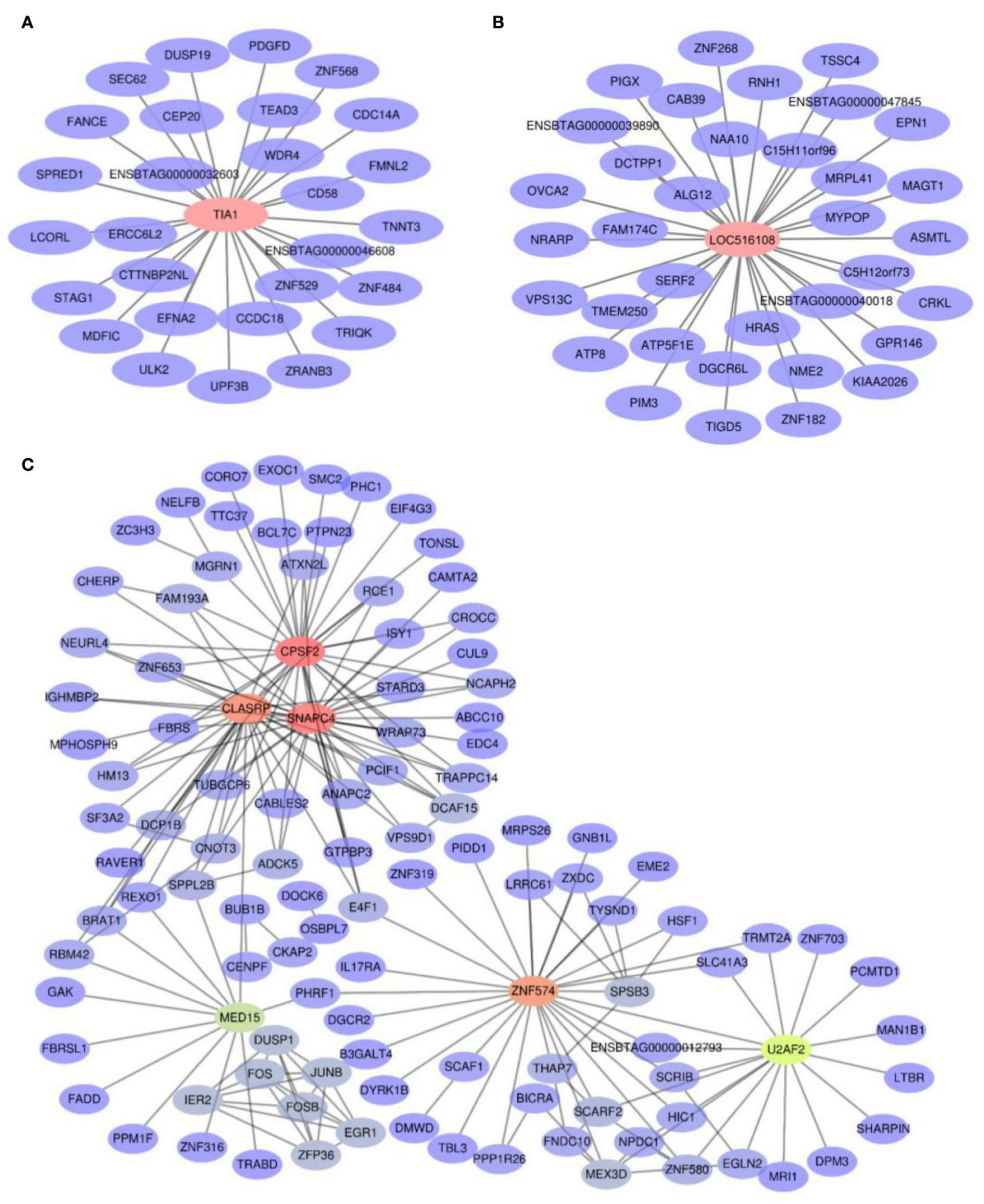
Co-expression network diagram of the interaction between hub genes and their target genes. **(A)** Grey60 module. **(B)** Magenta module. **(C)** Pink module.

To identify hub genes, | GS | > 0.2, | MM | > 0.9, and weighted *p* < 0.05 were used as the identification criteria in grey60, pink, and magenta modules (Supplementary Table S4). The *TIA1* gene in the grey60 module (Figure 5A) and the *LOC516108* gene in the magenta module (Figure 5B) met the requirements, which were exported to Cytoscape to construct a network of relationships between genes. The pink module had more genes, so the top 200 genes were selected according to weight, calculated, and visualized using the Cytohubba tab in Cytoscape (Figure 5C). The results showed that *TIA1, LOC516108, SNAPC4, CPSF2, ZNF574, CLASRP, MED15*, and *U2AF2* were hub genes.

### Expression analysis of hub genes

**Figure 6:**
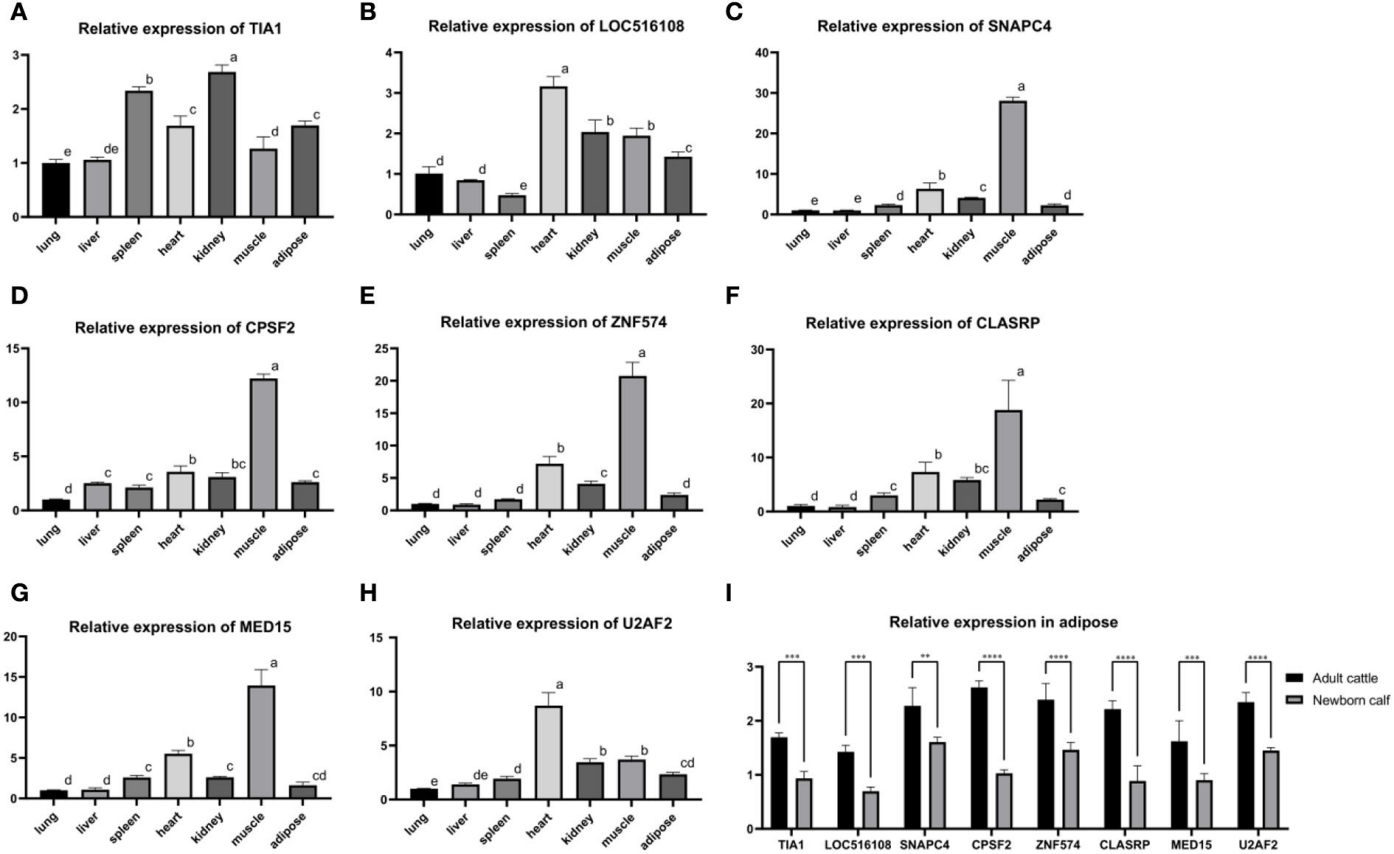
The tissue expression profile of 8 hub genes. **(A)** Tissue expression profile of *TIA1*. **(B)** Tissue expression profile of *LOC516108*. **(C)** Tissue expression profile of *SNAPC4*. **(D)** Tissue expression profile of *CPSF2*. **(E)** Tissue expression profile of *ZNF574*. **(F)** Tissue expression profile of *CLASRP*. **(G)** Tissue expression profile of *MED15*. **(H)** Tissue expression profile of *U2AF2*. Superscript letters indicate mean significant difference (*p* < 0.05). Values with superscript letters indicate a mean significant difference (*p* < 0.05). **(I)** Relative expression of adult cattle and newborn calf in adipose. *Denotes significance according to ANOVA methods, *p < 0.05, **p < 0.01, ***p < 0.001, and ****p < 0.0001.

RNA was extracted from the heart, liver, spleen, lung, kidney, muscle, and adipose tissue of the adult cattle and the adipose tissue of newborn calves, and reversely transcribed into cDNA. The primers of eight hub genes were combined with the tissue cDNA by PerfectStart Green qPCR SuperMix kit to determine the relative expression levels. The tissue expression profile showed that the relative expression level of eight hub genes was higher in adipose tissue as energy storage and muscle tissue as a metabolic organ (Figures 6A–H). At the same time, the expression level in adipose tissue of adult cattle was significantly higher than the expression level in adipose tissue of newborn calves (Figure 6I).

## Discussion

In this research, bioinformatics analysis techniques were used to correlate the metabolic gene expression levels of subcutaneous adipose tissue of Norwegian red heifers with various energy and protein diets, and to predict the regulated lipid metabolism hub genes and biological processes linked with energy intake. The results indicated that different energy intakes were involved in the metabolic process of the subcutaneous adipose tissue of Norwegian red heifers, while different protein intakes did not significantly affect the metabolic process, which was consistent with the results of previous studies; but intergene regulatory network and hub genes were not identified in previous studies (39). Consequently, we eventually identified eight hub genes in three modules that regulate subcutaneous adipose tissue metabolism by weighted gene co-expression network analysis, laying a foundation for further understanding the regulatory mechanism of diverse energy intake on subcutaneous adipose tissue metabolism.

### Enrichment analysis results of three modules

GO functional annotation and KEGG enrichment analysis were carried out on the three selected modules, and the GO results showed that all 138 genes of the grey60 module were used as components of cellular nucleoplasm, and studies showed that part of the eukaryotic ribosome synthesis is carried out in nucleoplasm, resulting in huge energy consumption (40). Chromosome rearrangement in the nucleoplasm is also associated with carbohydrate intake (41), and energy intake may affect the biological processes in the nucleoplasm, thus affecting the expression of genes in the nucleoplasm. In addition, genes significantly associated with low energy and low protein in the grey60 module were primarily enriched in Rap1, MAPK, pancreatic secretion, glycerophospholipid metabolism, and so on. Studies have shown that the Rap1 signaling pathway controls systemic metabolism in the hypothalamus (42), regulates metabolic processes inside and outside the nucleus (43), and regulates energy dissipation processes such as plasma membrane transport signal transduction, endocytosis, exocytosis and cell membrane fusion (44). It has also been found to regulate the glucose metabolism process in mice, which can improve blood glucose and diabetes (45), and Rap1 protein is also an activator of MAPK signaling (46). MAPK signaling pathway is believed to play an important role in the regulation of insulin secretion and type II diabetes mellitus (T2DM) (47), and the secretion dose of insulin controls the lipid accumulation of precursor adipocytes and regulates the metabolism of adipose tissues (48). Besides, the MAPK signaling pathway was also found to be negatively regulated by TREM-2 in diet-induced diabetic mice (49), and genes enriched in the MAPK signaling pathway were shown to be related to lipid metabolism in mice (50). Because the main function of the pancreas is to secrete lipase (51), and the content of hydrolyzed fat of secreted pancreatic lipase accounts for more than 80% of the total diet (52), the pancreatic secretion process was markedly influenced. When mice were fed with various energy diets, the glycerophospholipid metabolism pathway was substantially distinct between low-energy and high-energy experimental groups (53), which was consistent with the results of this research. Therefore, GO and KEGG results revealed that genes in the grey60 module related to low energy and low protein diets were predominantly used as nucleoplasm components to regulate the adipose metabolism pathway.

In the GO results of the pink module, genes were mainly involved in RNA metabolism, regulation of nucleobase compound metabolic process, regulation of transcription, and regulation of nucleic acid-templated transcription as nucleoplasm, cytoplasmic, and organelle components. The KEGG analysis demonstrated that the module genes were mostly enriched in Rap1, MAPK, Notch, VEGF, IL-17, and GnRH signaling pathways. Rap1 and MAPK signaling pathways were also the main gene enrichment pathways in the grey60 module, which further confirms the importance of these two pathways for energy metabolism. Notch signaling was activated in mice fed a high-energy diet (54), and KCTD10 has also been recognized as an upstream regulator of Notch signaling to regulate brown fat thermogenesis and whole-body metabolism (55). Studies have also found that the VEGF signaling pathway is regulated by calcium dobesilate (CAD) to alleviate diabetes in mice with high energy diet (56), and gene encoding cyclooxygenase 2(COX2) regulates glucose and lipid metabolism by regulating VEGF signaling pathway in mice with obesity caused by high energy diet (57). Besides, studies have discovered that IL-17 and Azgp1 interact with each other to alter lipid metabolism in mice with a high-energy diet (58). Finally, gonadotropin has been found to be too low in rabbits on a high-energy diet (59), and metabolic pathways regulate the gonadotropin signaling pathway by affecting the hypothalamus have also been confirmed (60). In conclusion, the pink module is still closely associated with specific energy metabolism.

For the GO annotation, magenta module genes are mainly involved in carbohydrate metabolism as nucleoplasm, cytoplasm, and organelles, as well as KEGG, showed that genes in the module were enriched in thermogenesis, oxidative phosphorylation, pyrimidine metabolism, insulin signaling pathway, adipocytokine signaling pathway, AMPK signaling pathway, metabolic pathways, and VEGF signaling pathway. In the clinical study, 28 people were put on a low-energy diet, and the blood analysis of the patients indicated that a low-energy diet involved carbohydrate metabolism and insulin secretion (61). Insulin secretion and AMPK signaling pathway were also considerably modified after 14 weeks of high and low-energy diets in mice (62) in the magenta module. In previous studies, carbohydrate metabolism in adipose tissue was altered when mice were fed diets with different energy levels (63), and modulation of energy levels in the rat diet was also found to result in shifts in AMPK and insulin signaling (64). Likewise, in previous studies, 3-month-old mice were treated with high-energy and low-energy diets for 72 h, respectively, and both treatments involved the insulin secretion process and VEGF signaling pathway (65). All these results prompted us to further evaluate the relationship between genes and changes in energy metabolism.

### Hub genes in three modules

Under the low-energy and low-protein diets, genes were down-regulated in the grey60 module. The *TIA1*, as the hub gene in the module, shuttled in the nucleus and was responsible for gene transcription and pre-mRNA splicing (66). In addition, the *TIAI* as an RNA-binding protein performed a role in translational regulation in the cytoplasm (67, 68), which is closely related to biological processes such as cell proliferation and apoptosis (69), immunity, and inflammation (70). At the same time, the *TIA1* gene has also been proven to be the core regulatory gene of RNA metabolism (71) and involved in a variety of cellular metabolic processes (72). It was identified that deletion of the *TIA1* gene in mice was comparable to mice under starvation conditions, leading to upregulation of *Plin4, Pnpla2, Pnpla7*, and other genes (73), which are responsible for lipid droplet generation (74, 75), free fatty acid supply (76), regulation of energy metabolism, and lipid metabolism (77). The downstream gene regulated by *TIA1* and *PDGFD* is also a newly identified adipokine (78) which is down-regulated during adipogenesis in humans and mice (79), consistent with the results of this analysis.

Genes in the pink module are positively correlated with low-energy and low-protein diets, and negatively correlated with high-energy and low-protein diets. Among the hub genes, *SNAPC4* is related to pancreatic development (80), and the functions of the pancreas are secreting digestive enzymes and hormones to coordinate the digestion and absorption of nutrients and energy metabolism (81, 82). The *BUB1B*, as their downstream target gene, is co-regulated by *SNAPC4*, the recognition-specific polyadenylation signaling gene (83, 84), is co-regulated by *SNAPC4, CPSF2* and the RNA splicing gene (85). The *CLASRP* and its expression fluctuated in the lungs of 16-week-old mice on a high-energy diet (86, 87). Moreover, the down-regulation of *BUB1B* gene expression was also determined after 72 h of OE33P cells in a high-fat medium (88). Down-regulation of *IL17RA*, a downstream gene of *ZNF574* (the hub gene in the pink module), was found to reduce the side effects of obesity in mice fed with high energy diet for 9 weeks (89). As a key factor in the lipid regulation (90), *MED15* converts saturated fatty acids into unsaturated fatty acids to regulate lipid metabolism (91). Its downstream target gene *FADD* has also been convinced to be a key factor in glucose and lipid metabolism (92). In addition, mice, after 15 weeks of high-energy feeding, were found with down-regulated *FADD* and were not as obese as wild-type mice (93), which confirmed that the body may affect metabolism through down-regulation of *FADD* and decrease the impact of obesity. The *U2AF2*, the last hub gene in the pink module, binds to the *U2AF1* (94) and regulates translation through RNA in the cytoplasm (95, 96). Down-regulation of its downstream gene *EGLN2* was found to ameliorate metabolic problems in mice fed a 12-week high-energy diet (97).

*LOC516108*, the hub gene in the magenta module, is a protein-encoding gene, and its regulated *CAB39* was found to be a direct target of microRNA-451 in adipocytes (98). After 20 weeks of high-energy feeding, microRNA-451 was down-regulated and *CAB39* expression was also altered in mice compared with the control group (99). The *ATP8* downstream of the hub gene principally affects mitochondrial function (100), and has been identified to regulate insulin secretion and glucose metabolism of pancreatic β-cells in high-fat diet mice (101). Other studies have found that *HRAS* is up-regulated in low-fat diet mice (102), which is also the gene downstream of *LOC516108*, mainly blocks fat generation and regulates energy metabolism (103, 104).

To further verify the reliability of the results, real-time quantitative analysis of eight hub genes was performed in the heart, liver, spleen, lung, kidney, muscle, and adipose tissue of healthy adult cattle and newborn calves. Compared to the eight hub genes in the relative expression of the adult cattle group, we found that the relative expression quantity was elevated in the adipose tissue of energy storage and muscle tissue as a metabolic organ. When adult cattle and calf adipose tissues were compared, the relative expression levels of the eight hub genes were considerably higher in adult cattle than in newborn calves and were two to three times higher numerically. These results suggest that the above eight hub genes can turn on the homeostatic regulation of the metabolism of substances in the body by adjusting the external feeding method. However, the regulatory mechanism of their metabolism *in vivo* is still unclear and needs to be further explored.

## Conclusions

In summary, we explored the effects of distinct energy and protein feeding methods on the changes of the entire transcriptome of cattle and screened out three related modules (grey60, pink, and magenta modules) by constructing a weighted co-expression network. They were related to the nucleoplasm, cytoplasmic, and organelle components, and participated in Rap1, MAPK, AMPK signaling pathways, and so on. Furthermore, we identified eight hub genes from these three modules, namely TIA1, LOC516108, SNAPC4, CPSF2, CLASRP, ZNF574, MED15, and U2AF2, which were all related to metabolic regulation. Our findings systematically elucidated the biological processes and important regulators closely related to subcutaneous adipose tissue metabolism, which would contribute to a better understanding of molecular mechanisms in the subcutaneous adipose tissue metabolism and provide useful reference information for molecular breeding of cattle.

## Data availability statement

The original contributions presented in the study are included in the article/Supplementary materials, further inquiries can be directed to the corresponding author/s.

## Ethics statement

The procedures for animal handling for experiments were approved by the Committee of Experimental Animal Management at Northwest Agriculture and Forestry University, China (protocol number: NWAFUCAST2018-168). Moreover, all applicable rules and regulation of the organization and government were followed regarding the ethical use of experimental animals. Written informed consent was obtained from the owners for the participation of their animals in this study.

## Author contributions

XW: conceptualization, investigation, data curation, and manuscript—original draft and revised. JW: conceptualization, investigation, and manuscript–revised. LZ: conceptualization, validation, writing—review and editing, funding acquisition, project administration, and supervision. SR: conceptualization, validation, and manuscript—review and editing. AA, HA, JD, and JM: validation, part of the data analysis and processing, and search for data and literature. XQ and SY: methodology and animal testing. DZ: methodology and use of analysis software. All authors have read and agreed to the published version of the manuscript.

## Funding

This research was funded by the National Key Research and Development Program of China National Natural Science Foundation of China (31972994), National Beef and Yak Industrial Technology System (CARS-37), Key Research and Development Program of Shaanxi Province (2022NY-050 and 2022ZDLNY01-01), and Special Project for the Central Government to Guide Local Science and Technology Development (2060404-51301).

## Conflict of interest

The authors declare that the research was conducted in the absence of any commercial or financial relationships that could be construed as a potential conflict of interest.

## Publisher's note

All claims expressed in this article are solely those of the authors and do not necessarily represent those of their affiliated organizations, or those of the publisher, the editors and the reviewers. Any product that may be evaluated in this article, or claim that may be made by its manufacturer, is not guaranteed or endorsed by the publisher.
